# A Role for Exosomes in Craniofacial Tissue Engineering and Regeneration

**DOI:** 10.3389/fphys.2019.01569

**Published:** 2020-01-14

**Authors:** Lyndon F. Cooper, Sriram Ravindran, Chun-Chieh Huang, Miya Kang

**Affiliations:** College of Dentistry, The University of Illinois at Chicago, Chicago, IL, United States

**Keywords:** exosome, oral, tissue engineering, bone, dentin, cartilage

## Abstract

Tissue engineering and regenerative medicine utilize mesenchymal stem cells (MSCs) and their secretome in efforts to create or induce functional tissue replacement. Exosomes are specific extracellular vesicles (EVs) secreted by MSCs and other cells that carry informative cargo from the MSC to targeted cells that influence fundamental cellular processes including apoptosis, proliferation, migration, and lineage-specific differentiation. In this report, we review the current knowledge regarding MSC exosome biogenesis, cargo and function. This review summarizes the use of MSC exosomes to control or induce bone, cartilage, dentin, mucosa, and pulp tissue formation. The next-step engineering of exosomes provides additional avenues to enhance oral and craniofacial tissue engineering and regeneration.

## Introduction

The reconstruction of craniofacial tissues embraces a broad set of biological and clinical interests. Whether the particular biological or clinical concern involves bone, cartilage, salivary glands, skin and mucosa, muscle or nerve, the fundamental principles and challenges of tissue regeneration are shared. Classically, tissue engineering efforts directed toward tissue regeneration involve three key elements. Scaffolds, growth factors and cells are used either separately or together in efforts to replace functional tissues (Langer and Vacanti, 1993). Regarding the cellular component of tissue engineering, mesenchymal stem cells (MSCs) have been the focus of many efforts for craniofacial regeneration. MSCs are widely available from diverse tissues and are capable of expansion (limited self-renewal) and multi-lineage differentiation into bone, cartilage, fat, muscle, and nerve (Pittenger et al., 1999). They do undergo eventual senescence and require significant time for expansion in culture. The use of allogeneic cells requires intensive screening to preclude disease transmission. Yet, unlike the use of embryonic stem cells or induced pluripotent stem cells, MSCs do not pose the same risk of teratoma formation following transplantation or bear the same ethical issues associated with ESC usen (Kim and Park, 2017). While tumor formation and chromosomal abnormalities have been reported in mouse studies, MSC performance in large animal studies or early phase clinical trials have not revealed tumorigeneses or ectopic tissue formation (Williams and Hare, 2011).

Mesenchymal stem cells may function in several ways following transplantation. Transplanted MSCs may engraft and differentiate to directly form new tissues, they may mediate host cell formation of new tissues by paracrine signaling to induce differentiated cells to direct tissue regeneration and angiogenesis, or they may control wound healing and regeneration by immunomodulatory regulation involving direct cell-to-cell or indirect secretory signaling. Immunomodulation is recognized as an important aspect of MSC function in wound healing and thus tissue engineering. These mechanisms may not be mutually exclusive and, importantly, local cues may influence tissue specific/lineage specific functions of the MSC in tissue regeneration (Gnecchi et al., 2005; Phinney and Pittenger, 2017). However, the paracrine MSC function may profoundly influence the success of MSC-based tissue engineering by affecting host stem cell protection, apoptosis, neo-vascularization, and recruitment and differentiation (Caplan and Dennis, 2006; Gnecchi et al., 2008). This underscores the advantages of engrafted MSCs as a source of sustained release of factors in response to local conditions.

Despite the reported advantages of MSCs, the clinical use of MSCs face pragmatic and regulatory hurdles. Expanding autologous cells is time-intensive and does not permit real-time therapeutics and expansion may exceed “minimal manipulation” guidelines, it requires screening of donors for allogenic cells, there are challenges of biobanking and cryopreservation, and the concept of potency and dosing of MSCs for regenerative therapies remains to be clarified (Liang et al., 2014).

**FIGURE 1:**
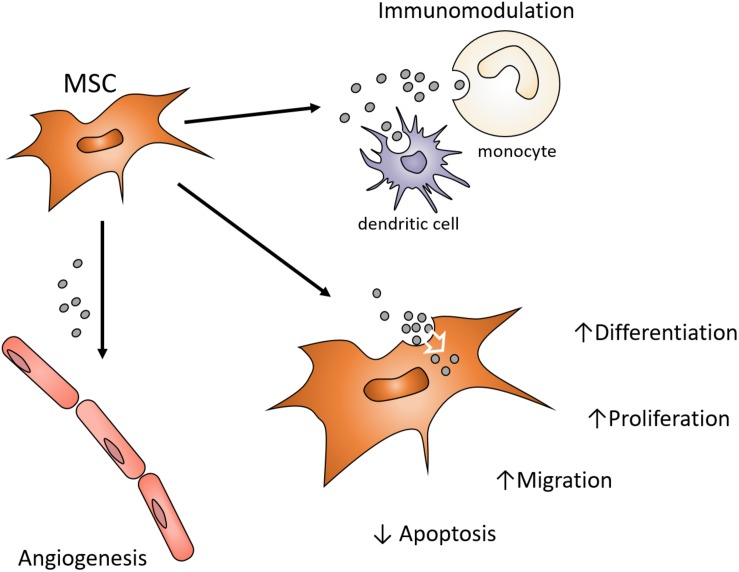
Possible MSC exosome functions contributions to paracrine signaling in Bone Regeneration. MSC exosomes endocytosed by various local cells can influence angiogenesis, inflammation and the functions of osteoprogenitor cells. Enhancing functions of endogenous cell types involved in craniofacial regeneration avoids the harvesting, expansion and use of autogenous or allogenic cells. Exosomes can provide alternative modulation of cell function to avoid use of growth factors or cytokines in order to alter target cell function.

Earlier studies of myocardial regeneration demonstrated that the conditioned media of MSCs could support myocardial regeneration *in vivo* (Gnecchi et al., 2005). While conditioned media has traditionally been considered a source of cytokines and growth factors supporting regeneration or lineage-specific differentiation, Lai et al. (2010) demonstrated that the exosomes of MSC conditioned media also positively influenced cardiac tissue regeneration/repair. Since then, there has been an expanding interest in the use of MSC exosomes as a cell-free alternative to MSCs for the purpose of directing tissue regeneration and tissue engineering (Phinney and Pittenger, 2017). Exosomes may exhibit functions attributed to the MSC that influence different target cells and their functions by controlling proliferation, differentiation, migration and apoptosis (Figure 1). MSC exosomes may present similar advantages, opportunities, and challenges the context of craniofacial regeneration (Pilipchuk et al., 2015). This review will consider the role of exosomes in tissue engineering and their potential use in craniofacial regeneration and repair.

## Exosomes Defined

**FIGURE 2:**
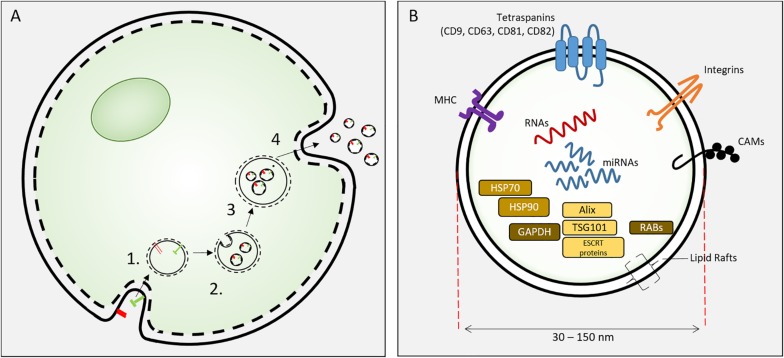
**(A)** Exososome biosynthesis. Exosomes are formed from endosomes (1) by an inward budding process to form intracellular vesicles (2). These mature as multivesicular bodies (3) that fuse with the cell plasma membrane to release exosomes. **(B)** Exosomes are 30–150 nm extracellular vesicles containing specific proteins, RNAs and lipids. Proteins include HSP70, 90, GAPDH; proteins involved in synthesis (Alix, ESCRT proteins, TGS101), and membrane associated or transmembrane proteins (RABs, Annexins, CAMs, Integrins, Tetraspanins, MHC I and II) and other cytosolic proteins.

Exosomes are specific extracellular vesicles (EVs) secreted into extracellular fluid (and culture media) by all cells. EVs include apoptotic bodies (500 nm–2 μm), microvesicles (100–1000 nm) and exosomes (30–150 nm). Exosomes were identified by Pan and Johnstone (1983) and were distinguishable from other extracellular vesicles by their biogenesis, size and biochemical composition. Exosomes are 30–150 nm vesicles derived from inward budding of endosomal membranes of multi-vesicular endosome (MVE) to form intraluminal vesicles (ILV). Fusion of the MVE with the plasma membrane results in the release individual exosomes (Kowal et al., 2014). These nanoscale lipid bilayer exosomes carry lipids, mRNA, miRNA and proteins derived from the parental cell (Figure 2). The biogenesis of exosomes involves the sorting of lipid-membrane associated proteins by ESCRT-dependent and –independent mechanisms, intracellular exosome trafficking and the endocytosis of exosomes by recipient (target) cells and this has been comprehensively reviewed (van Niel et al., 2018).

Exosomes of all cells are characterized by exosome-specific protein and lipid content. The exosome lipid bilayer is specific to EVs with noted enrichment in cholesterol, sphingomyelin, phosphatidyl serine and notable similarity to lipid rafts (Tan et al., 2013; Skotland et al., 2019). Exosomes contain plasma, cytosolic and nuclear proteins (Kowal et al., 2014). Several proteins are associated with exosome biogenesis (ESCRT proteins, Alix and TSG 101) and membrane function (RAB proteins, annexins, integrins, tetraspanin, MHC class II, CAMs). HSP70 and HSP 90 are common cargo proteins (Kalluri, 2016). In a proteomic analysis of MC3T3-E1 cell exosomes, 1069 proteins were identified and 786 overlapped with the current ExoCarta database. The associated pathway analysis revealed Integrin and mTOR signaling pathways, both of which are important in osteoblast differentiation and bone formation (Ge et al., 2015). Differences in EV protein content of MSCs from different sources implies unique functionality of EV from these different sources (van Balkom et al., 2019). A recent substantial review of the proteomic content of MSC EVs revealed that despite the different isolation methods and MSC sources, 44% of proteins are common to 7 of 10 datasets of EV proteins versus only 20.4% common MSC proteins compared to EV of non-MSCs. MSC exosomes, as currently isolated, may represent a heterogeneous population of nanoscale EVs that contain different protein cargo (Toh et al., 2018a); further development of isolation techniques may improve the ability to direct protein cargo of exosomes for therapeutic use. Studies focused on the MSC exosome proteins indicate that MSC exosomes may deliver specific proteins to control aspects of regeneration that include apoptosis (Lai et al., 2013), angiogenesis (Anderson et al., 2016), cell migration (Zhang et al., 2015) and lineage-specific cell differentiation (Wu et al., 2018). Anderson et al. (2016) noted that the proteome of MSC exosomes lack signaling proteins such as cytokines and growth factors, but instead contain downstream mediators of these pathways. Exosomal proteins may compliment the soluble growth factor/signaling of MSCs, thereby constituting a “dual-path” paracrine signaling mechanism of the secretome.

Significant effort has been extended to define the exosomes’ content (“cargo”) that is a result of exosome biogenesis involving direct and indirect targeting of cellular constituents. Exosomes contain protein, mRNA, miRNAs, and lncRNAs. Perhaps the most abundant component of the exosome cargo is miRNAs, non-coding RNAs (17–24 nucleotides) that regulate gene expression. MSC exosomes contain approximately 150 miRNAs (Lai et al., 2015) encoding regulators of signaling pathways involved in repair and regeneration (e.g., ERK, SMAD). ExoCarta^1^) is an exosome database providing data and statistics regarding exosomal cargo including identified miRNAs. A second open resource for EVs is Vesiclepedia^2^.

Exosome miRNA content is representative of the parental cell and indicates that exosome miRNA content is selective and appears to be specific to the exosome derived cell type and cell condition (e.g., hypoxia, inflammation). Furthermore, it is possible to direct miRNAs into exosomes using a specific EXO-motif (GGAG) (Villarroya-Beltri et al., 2013). Thus, miRNAs can be engineered to therapeutically alter cell functions. Exosome miRNA cargo specificity may also be diagnostic. While less abundant, lncRNAs may be important as constituent exosomal cargo. lncRNA control of cell function in genetic reprograming implies that exosomal lncRNA may also function in exosome-mediated changes in target cell function (Conigliaro et al., 2017). The exosome cargo appears to be a specifically encapsulated mixture of proteins and RNAs that present valuable new opportunities for diagnostics and therapeutics.

## Exosome Function in Paracrine Signaling; Horizontal Transfer of Protein and RNA

Exosomes were initially considered carriers of insignificant cellular debris. The current study of exosomes as mediators of cell signaling was advanced by Raposo et al. (1996), Zitvogel et al. (1998), and Raposo and Stoorvogel (2013) who demonstrated that lymphocyte exosomes carried MHC class II antigens from cells to the extracellular fluid and induced a MHC class II T cell response, thereby suggesting that exosomes serve to convey signals from cell to cell. Subsequently, Valadi et al. (2007), showed that exosomes contained mRNA and miRNA that was shuttled by exosomes from one cell to another. Additional studies have demonstrated that miRNA transport to target cells by exosomes results in miRNA-mediated alteration in gene expression and cellular function. Importantly, exosomes mediate the horizontal transfer of mRNA from the parental cell to the target cell and imply an important paracrine signaling mechanism previously not understood (Tomasoni et al., 2013).

There exists substantial evidence that miRNA cargo participates in exosome-mediated paracrine signaling. Using Drosha knockout mice that have demonstrable inability to produce miRNAs, Collino et al., demonstrated that the MSCs of Drosha KO mice with Acute Kidney Injury and the MSCs depleted of Drosha were unable to activate regenerative gene expression in injured kidneys. Implied is that the exosome miRNAs function in the paracrine signaling of regeneration (Collino et al., 2015). Argonaut 2 (Ago2) binds to miRNAs to generate Ago2-miRNA complexes that are sorted to exosomes (McKenzie et al., 2016) and serves to cleave targeted mRNAs. The knockdown of Ago2 in MSCs rendered the MSC exosomes incapable of promoting cortical axonal growth compared with native MSC exosomes (Zhang et al., 2017). This study further demonstrated that miR-17-92 cluster-enriched exosomes enhanced axonal growth, demonstrating that modified miRNA exosome content alerted a specifically targeted cellular response. Indirectly, CD9-/- mice produce fewer exosomes and displayed diminished bone fracture healing (Furuta et al., 2016). These different studies all implicate exosome miRNA in mediating MSC exosome paracrine signaling.

The signaling by exosomes can serve regenerative therapy (Figure 2). Exosomes are able to alter cellular activities related to: epithelial/mesenchymal transition, matrix remodeling, apoptosis, cellular differentiation, inflammation and repair processes (Guo et al., 2017). To date exosome administration has been associated with the prevention of apoptosis, promotion of proliferation, directing differentiation and the promotion of neovascularization. Thus, exosome-mediated regeneration has become a topic of intense interest in several fields.

Recent reviews highlight the potential of exosomes to direct regeneration, to further manipulate exosome cargo and target exosomes to target cells and to formulate specific modes of delivery to damaged cardiac tissues (Shanmuganathan et al., 2018; Zamani et al., 2018). Bruno et al. (2009) who used MSC exosomes to reduce experimental kidney damage also revealed early evidence of exosome function in regeneration. Neurological and ophthalmological fields are active in the exploration of exosomes in regenerative therapy. In a rat model, intravitreal MSC exosome treatment preserved retinal ganglion cell function and promoted regeneration of axons. Importantly, the effects were mitigated by knockdown of Ago2, a central regulator of miRNA function, suggesting that MSC exosome effects are miRNA dependent (Mead and Tomarev, 2017).

The regeneration of other tissues is now being studied in the context of exosome function and therapeutics. For example, a role for specific exosomal miRNAs in nerve regeneration has demonstrated the favorable modulation of nerve injury (Chen et al., 2019). MSC exosomes may function as regulators of skeletal muscle regeneration. The administration of MSC exosomes to C2C12 cells induced MyoD and Myogenein gene expression and administration of MSC exosomes following cardiotoxin-induced muscle injury resulted in enhanced muscle regeneration as demonstrated by greater diameter and numbers of myofibers, enhanced angiogenesis and reduced fibrosis (Nakamura et al., 2015). In studies involving nerve cell, macrophage, and MSC derived exosomes, all appear to influence inflammation, apoptosis, proliferative and differentiation/axonal outgrowth during peripheral nerve regeneration (Qing et al., 2018).

The control of angiogenesis is central to all regenerative and wound healing efforts (Park and Gerecht, 2014) and important observations have been made regarding the potential utility of MSC exosomes to promote angiogenesis. MSCs influence angiogenesis by secretion of paracrine factors (Watt et al., 2013); today exosomes are regarded as influential members of the MSC secretome. MSC exosomes may promote angiogenesis required for regenerative strategies affecting diverse target tissues. For example, MSC exosomes transfer miRNAs to HUVEC cells to promote tube formation and endothelial cell mobilization (Gong et al., 2017). However, MSC exosomes can exert both pro-and anti-angiogenic effects influencing VEGF expression. Further ROS and hypoxia influences on MSCs can alter the exosome cargo and exosome influence on angiogenesis (Alcayaga-Miranda et al., 2016). For these reasons perhaps, MSC-derived exosomes have been successfully used in numerous studies of regenerative medicine (Codispoti et al., 2018).

## MSC Exosome Roles in Oral and Craniofacial Tissue Engineering

Exosomes harvested from MSCs derived from oral tissues may be isolated and used for craniofacial tissue engineering. Exosomes from diverse tissues may also be used to promote craniofacial repair and regeneration (Table 1). The potential use of exosomes for craniofacial and oral tissue regeneration embraces bone tissue engineering. MSC exosomes have be used to enhance fracture healing in mice. Importantly, when CD9−/− mice that display reduced exosome production and display retarded callus formation were treated with exosomes, CD9−/− mouse fracture healing was accelerated (Furuta et al., 2016). Mechanistic studies suggest that exosome miRNAs regulated osteoblastic differentiation. Specific pathways including Wnt pathway and PI3K/Akt pathways may be affected by MSC exosomes (Hao et al., 2017).

**TABLE 1:** MSC exosomes in craniofacial repair and regeneration.

**Tissue**	**Exosome source**	**References**	**Type of study**	**Observation**
Bone	Bone marrow MSC	Cui et al., 2016	*In vitro*	Mineralizing osteoblast exosomes induce osteogenic differentiation of precursors
		Narayanan et al., 2016	*In vitro*	Differentiating MSC exosomes induce osteogenic differentiation of naïve MSCs
	Dendritic cells	Wang Z. et al., 2014	*In vitro*	Dendritic cell exosomes trigger MSC osteogenic differentiation
	Adipose MSC	Li W. et al., 2018	*In vivo*, mouse calvarial defect model	Tissue engineered bone graft doped with exosomes induces bone regeneration
		Lu et al., 2017	*In vitro*	TNFα-preconditioned MSC exosomes induce osteogenic differentiation of naïve MSCs
	Monocytes	Ekstrom et al., 2013	*In vitro*	Monocyte-derived exosomes promote osteogenic gene expression in MSCs
Cartilage	Bone marrow MSC	Zhang S. et al., 2018	*In vivo*, rat osteochondral defect model	MSC exosomes promote repair by attenuating apoptosis, enhancing proliferation and reducing immune reactivity
	Embryonic MSC	Zhang S. et al., 2016	*In vivo*, rat osteochondral defect model	MSC exosomes promote healing of rat trochlear groove osteochondral defect
	Chondrocytes	Chen et al., 2018	*In vivo*, rabbit progenitors + alginate scaffolds in nude mice	Chondrocyte exosomes promote subcutaneous stable ectopic chondrogenesis
Dentin/dental pulp	DPSC	Huang et al., 2016	*In vivo*, subcutaneous root slice model	Exosomes from differentiating DPSCs promote dentin/pulp regeneration in a subcutaneous tooth root slice regeneration model
Periodontal ligament	Bone Marrow MSC	Chew et al., 2019	*In vivo*, rat periodontal defect model	MSC exosomes improve periodontal ligament function and promote regeneration
	Adipose MSC	Mohammed et al., 2018	*In vivo*, rat ligature model	Adipose MSC exosomes can be used as a non-surgical adjunct treatment to improve periodontal repair and regeneration

Several studies have demonstrated that exosomes influence MSC osteoinduction. Exosomes from differentiating osteoblastic cells (Cui et al., 2016), dendritic cells (Wang Z. et al., 2014), and monocytes (Ekstrom et al., 2013) can potentiate MSC osteoblastic differentiation in cell culture. MSC exosomes and exosomes of MSCs undergoing osteoblastic differentiation also induce osteoblastic differentiation. In a study involving mineralizing MC3T3-E1 cells, 43 highly expressed miRNAs that, upon gene ontology and pathway network analysis, revealed their possible action in Wnt, Insulin, TGFβ, and calcium signaling pathways. Associated with increased target ST2 cell osteogenesis, mineralizing MC3T3-E1 cell exosomes inhibited Axin1 expression and increased β-catenin expression (Cui et al., 2016). Similarly, C2C12 cells expressed 12 miRNAs highly in isolated exosomes that promoted MC3T3-E1 cell osteoblast differentiation. When miR-27a-3p was depleted, targeted MC3T3-E1 cell exosome-dependent differentiation was prevented. A β-catenin dependent mechanism mediated this exosome (+miR-27a-3p) action in osteoblasts (Xu et al., 2018). MSC exosomes carry miRNAs that impact osteoblast physiology and bone regeneration. Several reviews have tabulated many miRNAs (primarily derived from osteoblastic cell lines and MSCs) presently implicated in control of osteoblast function and related bone regeneration (Papaioannou et al., 2014; Fang et al., 2015; Huang et al., 2017). In aggregate, these reviews demonstrate that MSC exosomes carry many miRNAs that impact key signaling pathways that include TGFβ, BMP, and Wnt pathways to influence SATB2, Runx2, Dlx5, and Osx function in control of osteoblast differentiation. Such studies indicate that exosomes modulate the physiology diverse target cells within regenerating bone tissue to influence the process of bone regeneration and repair.

The osteoinductive potential of MSC exosomes is apparently dependent upon the state of the parental cell. When human Adipose-derived MSC exosomes were obtained following osteogenic induction, the exosomes have greater osteoinductive effect. These exosomes accelerated bone healing in a calvaria defect model (Li W. et al., 2018). By pretreatment of Adipose-derived MSC with TNFα to mimic the acute inflammatory phase of wound healing, proliferation, migration, osteogenic gene expression and mineralization was increased in target hOB cell culture. This was attributed, in part, to increased Wnt3 within exosomes purified from TNFα treated hOB (Lu et al., 2017).

Exosomes from human iPS-MSC exosomes promoted osteoblast proliferation and differentiation in cell culture and, when applied with a βTCP scaffold for healing of rat calvaria critical-sized bone defects, the exosomes enhanced bone regeneration and angiogenesis (Qi et al., 2016). In addition, using human iPS-MSC exosomes, a dose-dependent increase in rat calvaria critical-sized defect bone regeneration was demonstrated by μCT and immunohistochemistry. In parallel, gene expression analysis implicated the activation of the PI3K/Akt pathway in iPS-MSC exosome treated BMSCs and this was affirmed using the Pi3K inhibitor LY294002 to block the effect of exosomes on osteogenesis (Zhang J. et al., 2016).

Cartilage repair and regeneration is affected by MSC exosomes and those of chondrocytes. The treatment of rat osteochondral defects with exosomes isolated from human MSCs (derived from HuES9 human embryonic stem cells) induced complete cartilage and subchondral bone repair at 12 weeks that was equivalent to unoperated control sites (Zhang S. et al., 2016). The authors indicated that many components of the MSC exosome were required for effective tissue regeneration and further suggested that exosomes represented an advantageous “cell-free” strategy for leveraging human embryonic MSCs for cartilage repair. Toh et al. (2017) reviewed the potential use of exosomes for induction of chondrogenic differentiation and upregulation of chondrogenic transcription factors (Toh et al., 2017). Further, it was demonstrated that MSC exosome-mediated cartilage repair also involved the specific immune modulation as demonstrated by the selective alteration of the local population of regenerative M2 macrophages versus pro-inflammatory M1 macrophages (Zhang S. et al., 2018). The potential to tailor exosome-mediated regenerative therapies to unique clinical conditions was recently demonstrated (Tao et al., 2017). They engineered human synovial MSC exosomes to carry increased levels of miR-140-5p with the successful purpose of directly increasing proliferation and migration of targeted chondrocytes without suppression of ECM protein synthesis. MSC exosomes may exert specific influences on different cells within the local environment to control regeneration.

When exosomes purified from human MSC conditioned media were applied weekly to healing of critical sized osteochondral defects in the distal femurs of Sprague Dawley rats, increased ICRS scores and O’Driscoll scores were notes with histological evidence of increased tissue repair. Exosome treatment over 12 weeks achieved regeneration hyaline cartilage and subchondral bone compared to fibrous repair in the PBS treated control limbs. The effect observed was attributed to multiple functions of exosomes including immunomodulation and an array of exosomal components required to orchestrate tissue regeneration of bone and cartilage (Zhang S. et al., 2016).

Additionally, chondrocyte-derived exosomes directed the experimental subcutaneous formation of cartilage-like tissues. It was suggested that maintaining a favorable chondrogenic “niche” was the result of antiangiogenic factors of chondrogenic exosomes (Chen et al., 2018). Many exosomes possess immunomodulatory function that is essential to the process of cartilage regeneration (Toh et al., 2017). This was reflected in a recent report of M2 macrophage polarization in healing osteochondral defects following MSC exosome treatment. In this study, increased cell proliferation, reduced apoptosis and enhanced matrix synthesis accompanied cartilage repair and regeneration (Zhang S. et al., 2018). These studies of cartilage repair indicate that exosomes are effective in directing tissue-specific regeneration that involves the targeting of multiple biological and cellular processes.

Exosome function is well defined in development (Hayashi and Hoffman, 2017) and has been convincingly demonstrated by example in the submandibular gland. Exosomal miRNAs transferred from mesenchymal cells to epithelial progenitors experience miRNA dependent epigenetic regulation of gene expression. Exosomes also function during reciprocal epithelium-mesenchyme interactions during tooth development. When Rab27a/b knockdown was used to disrupt exosome biosynthesis, enamel and dentin formation was reduced in organ culture experiments. Accordingly, the investigators further identified miR135a as an exosome component influencing Wnt signaling that increased dentin matrix protein expression (Jiang et al., 2017). Another recent study demonstrated exosome miRNA regulation of apoptosis in tooth development where the upregulation of miR133b – induced apoptosis in primary dentin mesenchyme and its ectopic expression prevented proper experimental tooth formation (Li Y. et al., 2018). Such studies implicate exosomes as key signaling vehicles in oral and craniofacial tissue development and suggest that exosomes may be utilized in their regeneration by control of apoptosis, proliferation, and differentiation of targeted cells.

Despite advances using exosomes in other fields of medicine, even the most recent general reviews of periodontal tissue regeneration have not considered that MSC exosomes have the potential to direct periodontal regeneration and repair (Chew et al., 2019). Adipose derived stem cell exosomes used to augment non-surgical periodontal therapy in a rat model of ligature-induced periodontitis positively affected periodontium regeneration (Mohammed et al., 2018).

## A Role for MSC Exosomes in Immunomodulation

Mesenchymal stem cells are widely distributed among tissues and appear to communicate with the local inflammatory microenvironments in the process of tissue repair and regeneration. It has been suggested that MSCs are involved in the recruitment and modulation of inflammatory cells during repair and regeneration. Following damage, recruited MSCs are known to produce immunoregulatory factors that contribute to the progression of inflammation and wound healing. The MSC secretome is known to contain many growth factors and cytokines that direct angiogenesis, extracellular matrix synthesis and remodeling as well as progenitor cell differentiation. With regard to immunomodulation, MSCs act by direct cell contact with immune cells and they secrete potent factors including TGF-β, NO, PGE2, 1L-17, and IL-10. MSCs communicate to alter TC, dendritic cell, B cell and NK cell activities to modulate inflammation during the repair process. While the immunomodulatory role of MSCs has been broadly reviewed (Wang Y. et al., 2014; Gao et al., 2016), a role for exosomes as part of the MSC’s immunomodulatory secretome has yet to be fully characterized.

MSC exosomes have been examined in the context of immunomodulation. The potential mechanisms of immunomodulation by MSC exosomes include their impact on tissue resident macrophages that includes suppression of M1 macrophage polarization and enhancement of M2 macrophage activation. MSC exosomes achieve this alteration of M1/M2 ratios in healing tissues by directly targeting signaling pathways influencing macrophage polarization. Beyond the macrophage, MSC exosomes also influence dendritic cells, B cells, and NK cells of the immune system. The prominent effect of MSCs on T cell function is well known and several reports indicate that MSC exosomes can modulate T cell function (Seo et al., 2019). It has been noted that tissue macrophages may vary in their influence on immunomodulation. It remains to be determined if exosomes of MSCs from various craniofacial tissues (e.g., gingiva versus dental pulp vs. alveolar bone) possess unique immunomodulatory functions.

When MSC exosomes have been studied in therapeutic animal models of cardiac infarction, liver disease, ischemic stroke brain injury, or induced kidney disease, the investigators have reported altered or reduced immune cell invasion into sites of injury or a decline in pro-inflammatory reactions (Borger et al., 2017). MSC exosomes appear capable of modulating both humoral and cellular immune responses (Toh et al., 2018b). Several clinical trials currently underway involve MSC exosomes for the treatment of type I diabetes, for chronic kidney disease, for macular degeneration and for ischemic stroke (Mendt et al., 2019). It is feasible that MSC exosomes can be applied clinically for local control of inflammation to enhance craniofacial regeneration or suppress disease.

The possible advantages for using MSC exosomes for immunomodulation include overcoming disadvantages of using cell-based therapies (e.g., variability in cell isolation and expansion, dosing, targeting) as well as being able to produce large numbers of exosomes from highly characterized parental cells or cell lines. In a translational effort, Toh and colleagues recently demonstrated that human MSC exosomes enhanced periodontal ligament cell migration and modestly enhanced periodontal regeneration as assessed by μCT and histological methods (Chew et al., 2019). In a similar manner, MSC exosomes enhanced repair of osteochondral defects that was associated with a reduction in proinflammatory cytokines and increased numbers of regenerative (M2) macrophages (Zhang S. et al., 2018).

## Exosome Isolation From Dental Tissue

**FIGURE 3:**
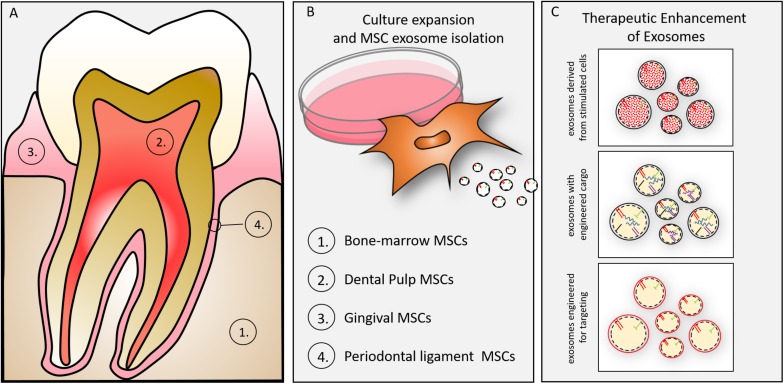
**(A)** Oral tissue-specific cell types are sources of unique exosomes. Exosomes have been isolated from cells of (1) bone, (2) dentin/pulp, (3) gingiva, and (4) periodontal ligament. These cell-type specific exosomes may provide lineage specific direction to craniofacial tissue engineering. **(B)** Culture expansion and MSC exosome isolation. MSCs can be isolated from many oral/craniofacial tissues and expanded in culture. MSCs have been isolated from bone marrow, dental pulp, gingiva and periodontal ligament. **(C)** MSC derived exosomes can be enhanced for therapeutic advantage by several techniques including (1) parental cell stimulation (e.g., growth factor, culture conditions), (2) engineering to direct inclusion of overexpressed miRNAs, (3) engineering to include membrane proteins (e.g., integrins) to target exosomes to tissues (or cells).

Exosomes may be isolated from MSCs of dental tissues (Figure 3). Either exfoliating deciduous teeth or intentionally harvested adult teeth can provide dental pulp stem cells (DPSCs) for regenerative purposes (Gronthos et al., 2000). DPSCs direct regeneration by *in vivo* multipotential differentiation, including liver, dental pulp, bone, muscle and nerve, besides promoting vascularization of the regenerating tissues (Gandia et al., 2008; Ikeda et al., 2008; Yamada et al., 2011; Bianchi et al., 2017; Zordani et al., 2019). DPSCs of neural crest derivation may provide particularly valuable function in nerve regeneration as demonstrated by their phentoype (Carnevale et al., 2018), their differentiation along neurogenic lineages and functional studies demonstrating DPSC-mediated functional regeneration of siatic nerve (Ullah et al., 2017). A primary limitation of DPSCs is the relatively small number of cells obtained for autologous use in regenerative medicine (Tatullo et al., 2015), yet they have been advocated as a suitable MSC for tissue/cell banking (Lizier et al., 2012). A primary advantage of the DPSC versus the bone marrow derived MSC is the relative availability and ease of access (extraction/endodontics). Our efforts to explore DPSC exosomes may provide an allogenic resource for regeneration using culture expanded or immortalized DPSCs. In light of the long-term stability of exosomes (Kalra et al., 2013; Lee et al., 2016) pulp derived exosomes may play a significant role in regenerative medicine. We and others, in preliminary cell culture and animal studies have affirmed that DPSC exosomes, like MSC exosomes promote immune-protective and anti-apoptotic mechanism in targeted cells and tissues (Jarmalaviciute et al., 2015) and, more specifically, DPSC exosomes demonstrate pulp regeneration potential (Huang et al., 2016).

Periodontal ligament cells have been an historical target source of multipotential stem cells to direct regeneration (Seo et al., 2004). Periodontal ligament stem cells (PDLSCs) have been isolated from the periodontal ligament (Zhu and Liang, 2015). PDLSCs differentiate along osteogenic, adipogenic, and chondrogenic lineages (Gay et al., 2007). Additionally, they are self-renewing when transplanted into ectopic sites and can differentiate to form mineralized structures *in vivo* (Menicanin et al., 2014). Although the regenerative properties of PDLSCs has been studied extensively, the PDLSC derived exosomes have not been studied as extensively as the bone marrow derived MSCs (BMSC) exosomes.

The documented immunomodulatory properties of PDLSCs (Wada et al., 2009) have been attributed to paracrine function (Rajan et al., 2016b) and this is likely mediated by PDLSC derived exosomes (Rajan et al., 2016a; Misawa et al., 2019). Furthermore, exposure of PDLSCs to lipopolysaccharide triggers the production of exosomes that have the potential to induce pro-inflammatory M1 polarization (Kang et al., 2018). The similarity of PDLSCs with the bone marrow derived MSCs in terms of basic characteristics raises interesting questions about their potential as a source of therapeutic exosomes in regenerative medicine applications.

Exosomes have also been isolated from gingival MSCs and their administration to tongue wounds increased BDNF expression and associated taste bud regeneration with neurofilament expression reflecting re-innervation (Zhang Y. et al., 2018). Gingival MSCs isolated from gingival lamina propria were used in a diabetes-induced wound closure assay to facilitated recalcitrant healing. When gingival MSCs in a chitosan/silk hydrogel were applied to cutaneous wounds in a rat model, the addition of gingival MSC exosomes enhanced wound closure rates. Both nerve density and microvessel density were increased in gingival MSC exosome treated wounds (Shi et al., 2017).

## Engineering Exosomes for Therapeutics

The innate characteristics of exosomes suggest innovative uses in clinical therapy. Their nanoscale size, membrane composition related to rapid endocytosis, relative lack of toxicity, stability, and access to the central nervous system (passing the blood brain barrier) combine to provide a vehicle for delivery of specific signals to target cells. These signals (protein, RNA, miRNA) represent lineage and physiology of the parental cell. Exosomes may also possess allogenicity enabling the use of well characterized and standardized donor sources. In addition, Xenographic success using human MSC exosomes to achieve positive results in animal studies has been repeatedly referenced in this review. However, the reciprocal xenograft studies have not been performed *in vitro* or *in vivo* and represent knowledge gaps that remain poorly understood.

**FIGURE 4:**
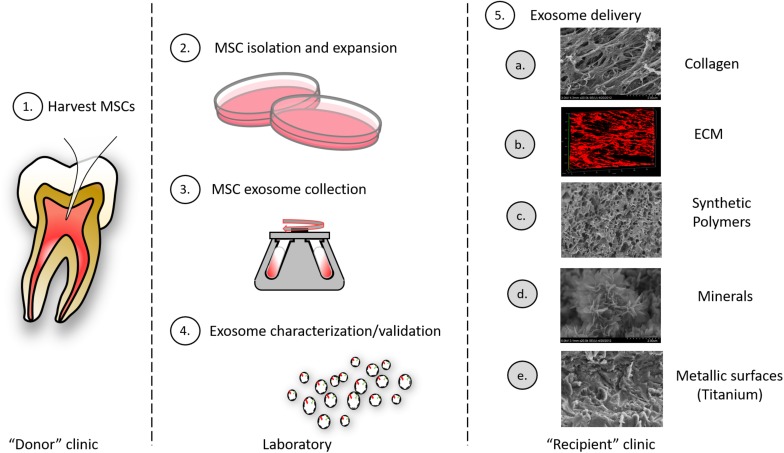
Exosomes as therapeutic agents: exosomes may be clinically isolated from MSC isolated oral tissues (e.g., perhaps by pulpectomy). (1) This could be an autogenous scheme or, alternatively, MSCs could be isolated from pulp of extracted teeth and derived exosomes used in an allogenic scheme. (2) MSCs must be isolated and expanded and conditioned media from cells collected. (3) MSC exosomes are collected from conditioned media by different methods including ultracentrifugation, immune-isolation, and precipitation. (4) Exosomes must be characterized according to their size and integral membrane protein composition; engineered exosome cargo must be defined by miRNA or protein analysis. (5) Exosomes can be delivered to a variety of different tissues using different carriers that include: (a) collagen matrices, (b) decellularized ECM, (c) synthetic polymers (bioprinted carriers), (d) mineral scaffolds, and (e) metallic implants.

Further, exosomes can be engineered to carry specified cargo and synthetic (pharmacologic) cargo for therapeutic intent (Arenaccio et al., 2019). Stimulation of, or the genetic manipulation of, parental cells can alter exosome cargo to enhance regeneration. Alternatively, selected siRNA, miRNA, drugs and enzymes can be loaded into exosomes directly by electroporation, chemical-based transfection, simple incubation methods (Johnsen et al., 2014; Liao et al., 2018). Early investigations also suggest that exosomes may be stored longer term, perhaps by lyophilization (Charoenviriyakul et al., 2018). Further engineering of exosomes can involve expression of surface proteins to direct exosomes to specific extracellular matrices or to selected cells (Figure 4).

Systemic administration of exosomes is challenged by the preferential uptake in liver, kidney and spleen or off target effects. Targeting of exosomes to cells or tissues may be accomplished by expression of proteins on the lipid bilayer (e.g., specific integrin, viral glycoprotein). For example, exosomes from parental cells engineered to an EGFR-specific peptide ligand were successfully targeted to xenograft EGFR^+^ breast cancer cells in mice (Ohno et al., 2013). Endogenous exosomal tetraspanin proteins may contribute the process of targeting. Targeting of exosomes was achieved by transfection of defined tetaspanin-integrins and intravenous injection of the engineered exosome resulted in preferential exosome uptake in the pancreas (Rana et al., 2012). Exosomal integrin involvement in exosome targeting was elegantly indicated by studies of exosomal localization in cancers where unique exosomal integrins interact with specific cell –associated extracellular matrix (Hoshino et al., 2015). To deliver siRNA in exosomes specifically to the brain, the central nervous system-specific rabies viral glycoprotein (RVG) was cloned in the integral exosome LAMP2b protein. The RVG engineered exosomes were specifically taken up by Neur02A cells. Following systemic injection of GAPDH siRNAs containing RVG exosomes in ice, significant brain tissue specific reduction in GAPDH mRNA expression was noted compared to other tissues of naked siRNA injection (Alvarez-Erviti et al., 2011).

These early investigations have yet to fully explore some of the challenges facing the use of exosomes as therapeutic agents in craniofacial regeneration. First and foremost, isolation methods are yet fully reproducible and scalable to meet expectations of good clinical practice. This embraces the matter of quality assessment of consistent isolation and integrity. Second, exosome stability and shelf life must be further explored; recent evidence suggests that lyophilization is a potential solution (Bosch et al., 2016; Charoenviriyakul et al., 2018). Third, delivery of exosomes requires careful consideration regarding targeting of specific cells and tissues, their use systemically and potential off-target complications. Delivery and targeting may be enhanced by engineering of the exosomes. Finally, the dosing (concentration and timing) and potency of exosomes must be carefully considered and methods of defining these clinical parameters must be addressed and adopted.

## Exosome Role in Oral and Systemic Disease Diagnosis

The role of salivary exosomes in diagnosing systemic and oral diseases is one example of current speculative applications for exosomes in medicine and dentistry (Han et al., 2018). The importance of saliva in the diagnosis of oral and systemic diseases is of growing interest. The field has expanded to use liquid biopsy (non-invasive biofluid tests) for analyses by proteomics, transcriptomics, microbiomics, metabolomics, and microRNAs. While not the intended focus of this review, exosome use in diagnosis of disease is of growing interest in medicine and dentistry and merits brief consideration here. Exosomes isolated from biofluids can overcome limitations such as relative low expression of biomarkers and degradation of RNAs and miRNAs in whole and glandular saliva. Exosomes can be isolated using commercially available kits, ultracentrifugation methods, and specialized aqueous two-phase system to improve salivary exosome isolation (Sun et al., 2016; Kim et al., 2017). Exosomes isolated from glandular saliva were shown to possess miRNAs unique to salivary gland disease status (Michael et al., 2010). The use of salivary exosomes as biomarkers for cancer has been recently reviewed (Nonaka and Wong, 2017). Characterization of intact exosomes by FTIR distinguished exosomes of Oral Cancer subjects from Healthy individuals and suggests a non-invasive method for future diagnosis of oral cancer in early stages (Zlotogorski-Hurvitz et al., 2019). Subsequent studies oral cancer (Economopoulou et al., 2017; Lousada-Fernandez et al., 2018), oral lichen planus (Byun et al., 2015), and Sjogren’s syndrome indicate the potential future use of salivary exosomes as diagnostic targets in liquid biopsy of oral and systemic disease.

## Summary

The past decade’s exploration of exosome biology has extended to consideration of exosome’s utility in controlling cell and tissue function during wound healing and regeneration. Significant progress has been made in understanding how exosome cargo is transferred to target cells and act as signals to alter cell function by means of packaged miRNAs and proteins. More recent advances in the engineering of exosomes demonstrate the possibility of generating cell free, nanoscale particles that can modulate specific cell functions and may target specific tissues and cells. The existing demonstration that MSC exosomes mediate the regeneration of bone, cartilage, dentin, muscle, blood vessels and nerves indicates that exosomes have possible application in oral/craniofacial regeneration. The role of MSC exosomes in immunomodulation is an important functionality that may have great importance in regeneration (and managing pathology) in the oral cavity. Given the stated advantages of exosomes when compared to other cell and molecular approaches to regeneration, exosome-based therapeutics may be of current importance in advancing oral/craniofacial regenerative therapy success.

## Author Contributions

LC conceived and performed the primary literature search of the document, and contributed to the writing and illustration of the manuscript. SR contributed to the writing of the document and illustrations. MK and C-CH contributed to the literature search and writing of the document.

## Conflict of Interest

The authors declare that the research was conducted in the absence of any commercial or financial relationships that could be construed as a potential conflict of interest.

## Footnotes

1 ExoCarta.org

2 microvesicles.org
